# Bidirectional EEG Neurofeedback of Sensorimotor Beta Oscillations Reveals Asymmetric Modulation and Strategy‐Dependent Learning

**DOI:** 10.1111/ejn.70681

**Published:** 2026-09-10

**Authors:** Krithika Anil, Stephen Hall, Jennifer A. Freeman, Jonathan Marsden, Giorgio Ganis

**Affiliations:** ^1^ School of Health Professions University of Plymouth Plymouth UK; ^2^ Brain Research and Imaging Centre, Faculty of Health University of Plymouth Plymouth UK; ^3^ School of Psychology University of Plymouth Plymouth UK; ^4^ DDRC Healthcare Plymouth UK

**Keywords:** beta rhythm, biomedical technology, brain computer interfaces, learning, neurofeedback

## Abstract

EEG‐based neurofeedback (ENF) may be utilised as an intervention for improvement of motor performance in Parkinson's movement impairment. This exploratory study aimed to determine the ability of healthy participants to bidirectionally modulate sensorimotor beta power using ENF in real time and examine learning trends. The protocol trained participants to increase and decrease the amplitude of their individual beta power over C3 using a double‐blind sham‐controlled and within‐subjects design. Fifteen healthy participants completed the study (mean age = 38.9, SD = 14.9, age range = 20–66; nine female and six male). Thirteen participants successfully decreased beta activity, three participants successfully increased it and two participants demonstrated bidirectional control. A Wilcoxon signed‐rank test showed that successful EEG control during real ENF was significantly higher than sham ENF when participants received the sham condition first (*Z* = 3.80, *p* < 0.001). When participants received the real condition first, the difference in success was not statistically significant (*Z* = 1.19, *p* = 0.233), indicating a transfer learning effect from real to sham sessions. ENF performance varied across ENF training based on modulation direction and cognitive variables, including mental strategy, engagement and fatigue. This study showed that initial ENF performance depends critically on how performance is defined and measured. Learning trends between real and sham conditions further suggest that early ENF performance primarily reflects strategy‐dependent, effort‐based control rather than stable neurophysiological plasticity. These findings highlight the need for a more precise framework for ENF evaluation, one that separates transient modulation, strategic learning and durable physiological adaptation.

## Graphical abstract

Healthy participants performed EEG‐based neurofeedback (ENF) to modulate sensorimotor beta activity. Most successfully decreased beta, few increased it and learning varied by strategy, engagement and fatigue. Early ENF performance reflects effortful, strategy‐driven control rather than stable neurophysiological change, challenging binary ‘responder”’ classifications.

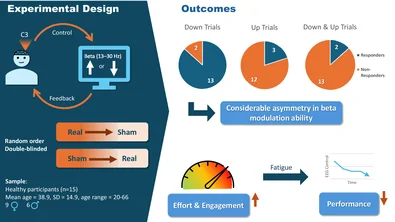

**Abbreviations**
BG: basal ganglia
CRED‐nf: Consensus on the Reporting and Experimental Design of clinical and cognitive‐behavioural Neurofeedback studies
EEG: electroencephalography
ENF: EEG‐based neurofeedback
ERD: event‐related beta desynchronization
ERS: event‐related beta synchronisation
fmaxβ: peak beta frequency
IC: imperative cue
IQR: interquartile range
MEG: magnetoencephalography
PSD: power spectral density
Rβ: individual beta range
thβD: ERD threshold
thβS: ERS threshold
USA: United States of America

## 1 Introduction

Parkinson's is a progressive neurological disorder that primarily disrupts motor functioning and affects 1–2 in every 1000 individuals in the general population (Tysnes and Storstein 2017). Motor symptoms in Parkinson's are believed to be driven by dopaminergic neurodegeneration in the basal ganglia (BG) (Radad et al. 2005), accompanied by elevated oscillatory activity across the motor network at beta frequency (15–30 Hz) (Eisinger et al. 2020), resulting in dysregulation of effective movement control (Silberstein et al. 2005). Current first‐line medications increase dopaminergic drive and provide symptomatic relief that is associated with a normalisation of beta power (Silberstein et al. 2005). Deep brain stimulation of the BG nuclei provides symptomatic improvements that are also accompanied by reduced beta power (Eusebio et al. 2012; Kühn et al. 2006). However, medication efficacy is variable and often accompanied by adverse side effects (Bronstein et al. 2011; Limousin and Foltynie 2019), greatly impacting quality of life for patients. These limitations highlight the need for adjunctive, noninvasive, non‐pharmacological approaches to complement existing treatments.

Oscillations are fundamental markers of communication within and between networks and are closely aligned with the coordination of effective function across cognitive and behavioural domains. In Parkinson's, patient studies recording from BG nuclei such as the subthalamic nucleus show that pathological elevation of these rhythms is associated with bradykinesia and akinesia (Brown 2007; Cagnan et al. 2019), with effective treatments, such as levodopa also reducing beta power (for a review, see Jenkinson and Brown [2011]). Importantly, the exaggerated beta oscillatory signatures are also robustly observed using noninvasive measurement of motor cortex activity (Pollok et al. 2013; Pollok et al. 2012). Similarly, these exaggerated motor cortex signatures are observed to be normalised by effective treatments in early‐stage Parkinson's (Hall et al. 2014; Prokic et al. 2019). Importantly, electroencephalography (EEG) and magnetoencephalography (MEG) studies have demonstrated that the movement‐related beta desynchronisation required for movement initiation is impaired in Parkinson's compared with controls (Deligani et al. 2025; Heinrichs‐Graham et al. 2014). These observations support the hypothesis that motor cortical beta is an anti‐kinetic signature and the inability to suppress exaggerated resting beta oscillatory power impairs effective initiation of motor programmes. Therefore, the development of effective, non‐pharmacological and non‐surgical approaches that increase the dynamic reactivity of motor cortex beta presents potential adjunct interventions for Parkinson's.

EEG‐based neurofeedback (ENF) uses real‐time modulation of cortical signals to control computer‐based feedback, most commonly visual stimuli (Marzbani et al. 2016), and may provide a valuable noninvasive means for individuals to modulate their own neural activity in real time. Despite decades of research, the clinical translation of ENF remains limited. A key barrier to progress is the lack of consensus regarding how ENF success should be defined, measured and interpreted. A recent systematic review examined factors underlying the slow development of the field and indicated that more rigorous and harmonised approaches may help unlock the therapeutic potential of neurofeedback in neurological disorders (Anil et al. 2021). This is consistent with broader work in the field of ENF development, such as Simon et al. (2021) who also emphasised that further studies are needed to optimise the time required to achieve effective ENF control and the challenges associated with sustaining cognitive strategies that reliably modulate adequately measurable EEG signals. In a subsequent blinded, sham‐controlled pilot study, our group showed that healthy participants could reliably reduce motor cortical beta synchrony, whereas attempts to increase beta synchrony were less consistent (Anil et al. 2024). These findings highlighted important barriers to advancing ENF as a treatment for PD. ENF performance was not yet sufficiently reliable within or across participants, and behavioural outcomes suggested that the bidirectional task imposed substantial demands that reduced success rates.

Emerging findings suggest that ENF performance may not be symmetric across directions of control. For example, Enz et al. (2022) demonstrated that healthy individuals can learn to bidirectionally modulate beta activity using ENF over the right inferior frontal cortex, with downregulation generally proving more successful than upregulation. However, whether similar modulation can be achieved over sensorimotor cortex remains unclear. Participants appear more able to reduce beta activity than to increase it, highlighting several issues that require clarification, including whether beta upregulation is intrinsically more difficult or mechanistically constrained, whether trial‐by‐trial switching between directions imposes additional task demands and whether ENF effects are too variable to support reliable control.

Although such approaches are ultimately intended for clinical populations, ENF protocols are inherently time‐intensive and cognitively demanding, which imposes a considerable participation burden. This is particularly relevant in Parkinson's, where fatigue and motor impairment may further constrain participation. Therefore, it is important to first establish robust, reliable definitions and determinants of ENF success in healthy participants under controlled conditions before translating these paradigms to patient groups.

To address these questions, the present study had two aims: (1) to determine whether healthy participants could achieve control of sensorimotor beta oscillations when modulation direction was tested independently and (2) to explore ENF performance and learning mechanisms that relate to the control of beta oscillations.

## 2 Methods

### 2.1 Participants

This study was designed as an exploratory preliminary investigation to examine neurofeedback success using our revised protocol and to explore patterns of neurofeedback learning. As the study was intended to generate preliminary estimates to inform a larger future study and not intended to formally test a hypothesis, a power calculation was not performed; this approach is consistent with recommendations from current literature on preliminary studies (Kunselman 2024; Leon et al. 2011; Misra et al. 2021). The target sample size was determined based on the objectives of the study and the aim of obtaining preliminary estimates of ENF success rates and effect sizes to inform the design of a future adequately powered randomised controlled trial. A target sample size of 20 participants was considered appropriate for the current context, which was informed by the literature (Kunselman 2024; Hertzog 2008) and comparable with our previous pilot study (Anil et al. 2024). Due to recruitment constraints and a lack of funding for participant reimbursement, 17 participants were recruited and a final sample of 15 participants were included in the study. This allowed detection of a 0.78 effect size (power = 0.8, alpha = 0.05).

Fifteen adult participants (mean age = 38.9, SD = 14.9, age range = 20–66; nine female and six male; 11 white British, two mixed white Asian, one Ceylonese and one Latino; all right‐hand dominant) were included in this study, recruited from the University of Plymouth via email advertisement using convenience sampling. Two additional participants were recruited, but their data were excluded from the study because one withdrew before study completion and the other produced unusable data due to excessive movements. Exclusion criteria included neurological and upper‐limb musculoskeletal conditions and medications impacting neural function.

Ethical approval was provided by the Ethics Committee from the Faculty of Health within the University of Plymouth (19/20‐1322). All participants were provided with an information sheet and had the opportunity to ask questions prior to the start of the study. Informed, written consent was taken from all participants before starting data collection. Participants were reminded of their right to withdraw from the study at any time without reason and were provided appropriate breaks through the study procedure.

### 2.2 Study Design and Protocol

This study used an updated protocol from our previous pilot study (Anil et al. 2024). The study was reported in accordance with the Consensus on the Reporting and Experimental Design of clinical and cognitive‐behavioural Neurofeedback studies (CRED‐nf) guidelines (Ros et al. 2020); the complete checklist is provided in the supporting information ‘SM ‐ NF_Reporting_Checklist’.

The current protocol used a double‐blind sham‐controlled, within‐subjects design. All participants completed both real and sham ENF tasks on separate days (sessions), with order randomised by computer. Each session comprised an eye‐open resting phase, an initial calibration Go/No‐go task, the ENF or sham task, a second Go/No‐go task and a final eye‐open resting phase.

The calibration and post‐Go/No‐go tasks were identical, each comprising 60 Go trials and 32 No‐go trials (Go/No‐go ratio = 1.875/1 ratio). The data from the calibration task were used to determine the individually specific beta frequency ERD maximum (fmaxβ) at C3, referenced to Cz. This peak frequency, ± 5 Hz (bounded between 14 and 31 Hz), defined the individual beta range (Rβ) used for all subsequent ENF and data processing (see EEG Methods). Defining Rβ from the calibration session ensured that the same frequency range was applied across both real and sham visits, minimising the influence of between‐session variability in EEG activity.

**FIGURE 1:**
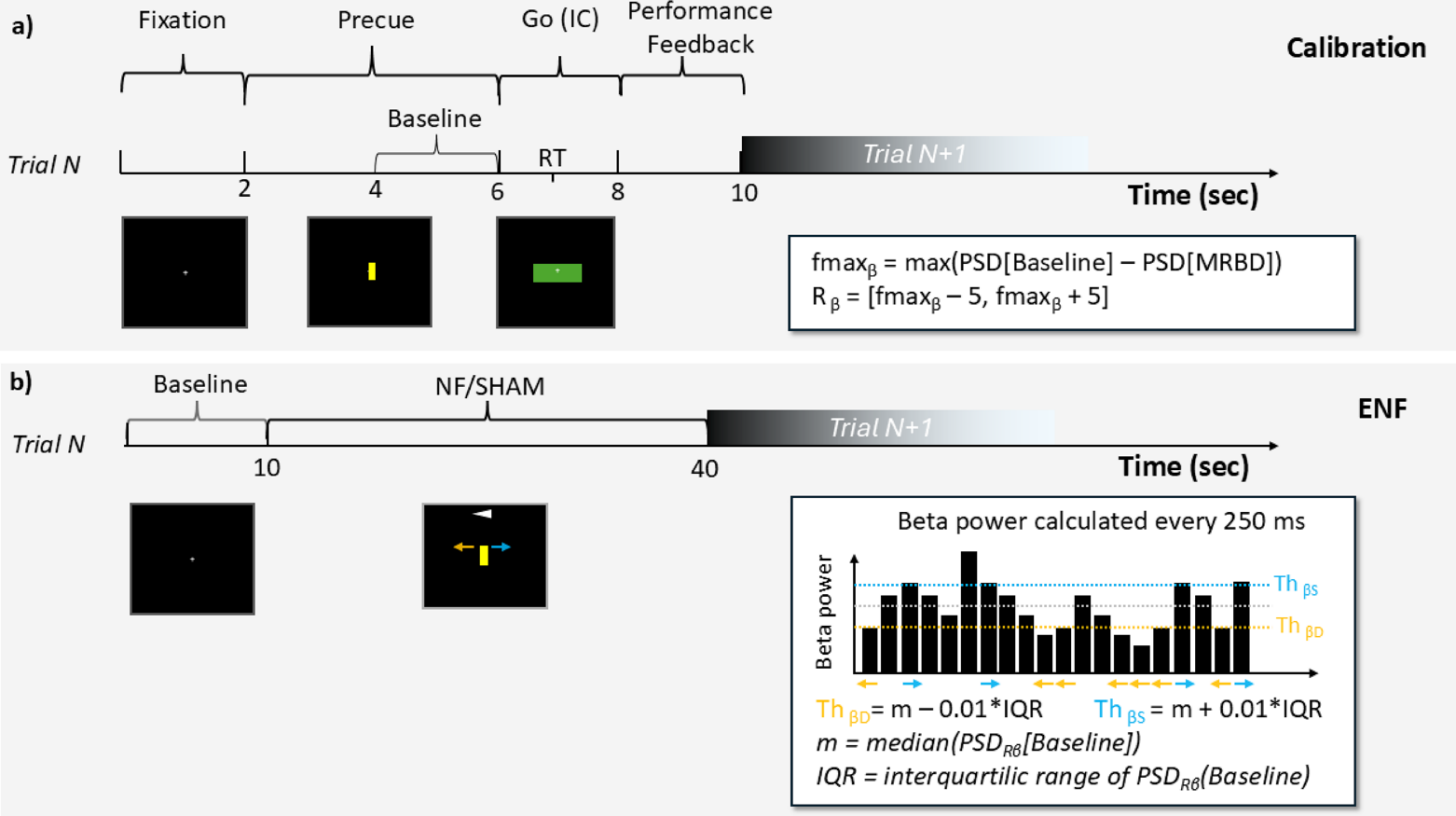
Trial structure (Go trial) and computations during the calibration (a) and EEG neurofeedback (ENF) phases (b). Timelines are schematic and not to scale. (a) Calibration. Each trial began with a fixation period, followed by a pre‐cue (stationary yellow bar at screen centre) and an imperative cue (IC; green rectangle on Go trials, red rectangle on No‐go trials). Participants responded as quickly as possible on Go trials and withheld responses on No‐go trials, after which performance feedback was presented. These data were used to verify beta‐band activity and estimate individual beta frequency parameters. (b) ENF. Each trial began with a baseline period, followed by a central yellow bar. A white directional arrow indicated the required movement direction (left, on the depicted trial). The bar position started at the centre, and it was updated in real time based on beta‐band power in the real condition, or based on replayed calibration data in the sham condition.

The Go/No‐go task required participants to execute or withhold a dominant‐hand index‐finger button press at imperative cue (IC) onset (Figure 1a). Each trial consisted of a central (2000 ms), followed by a yellow preparatory bar (4000 ms), and then the IC. The IC was either a green ‘Go’ rectangle (65%, 60 trials) or red ‘No‐go’ rectangle (35%, 32 trials). Participants were instructed to respond as quickly as possible on Go trials and to withhold responses on No‐go trials. Feedback was provided for slow responses (> 800 ms), anticipatory responses (100 ms) and commission errors. Correct Go trials were defined as responses occurring between 100 and 800 ms following IC onset.

In the subsequent ENF task, the yellow bar was presented with a directional arrow indicating the required movement. In the real condition, bar movement was driven by participants' real‐time EEG activity. In the sham condition, it was based on a replay of EEG recorded during the calibration task, providing a realistic control (see Figure 1b and section ‘Neurofeedback Presentation’ for processing details). Participants were instructed to move the bar in the indicated direction using mental strategies only and to avoid movement.

The task comprised six 6‐min blocks (54 trials in total; nine trials per block), with a short break between blocks. Participants completed a total of 18 min of training in one direction, followed by another 18 min in the opposite direction, with order counterbalanced across participants. During breaks, participants provided verbal ratings of task difficulty (‘how easy or difficult did you find moving the bar?’

Participants first completed an initial familiarisation block (5 min) without guidance. After this block, strategies were only suggested if participants requested them, with the emphasis that effective strategies vary across individuals and may be difficult to verbalise. The exact instructions were as follows: ‘neurofeedback can be an intuitive task. This means sometimes participants cannot describe what they are doing to make the bar move but they are still successful. Therefore, I would like you to try the first neurofeedback block without any suggestions and see if you can come up with a strategy yourself. After the first block, I will ask you whether you would like any suggestions that may help you. These suggestions are based on what has been reported by participants in previous research. They may or may not help you move the bar as everyone's successful strategy is different’.

Explicit instructions were not provided in the first block because there is currently no clear consensus within current literature regarding whether explicit cognitive strategy instruction facilitates or interferes with neurofeedback learning (Anil 2020; Kober et al. 2013; Lubianiker et al. 2022; Muñoz‐Moldes and Cleeremans 2020; Nan et al. 2012; Sitaram et al. 2017). Allowing an initial period of unguided exploration was intended to minimise researcher bias while enabling participants to develop their own strategies.

Following completion of the first neurofeedback block, participants who requested assistance were offered examples of mental states based on our first pilot study (Anil et al. 2024). These examples were drawn from a standardised list used for all participants and included a relaxed state, a stressed state, a focused state, or an emotional state. Mental states were suggested instead of strategies because our previous pilot study found that mental states were more useful to participants than specific mental strategies. For example, suggesting a ‘relaxed state of mind’ was more useful than suggesting to ‘imaging relaxing on a warm beach’. No participant‐specific guidance was provided.

### 2.3 EEG Methods

#### 2.3.1 Recordings

EEG was recorded using a 32‐channel actiCHamp Plus system (Brain Vision, North Carolina, United States of America [USA]). Data were sampled at 500 Hz. No online filters were used, and the EEG data + event streams were saved in .xdf format, reference‐free, via the Python actiChamp connector (https://github.com/brain‐products/LSL‐actiCHamp) and LabRecorder (https://github.com/labstreaminglayer/App‐LabRecorder) applications. Incoming EEG data were processed in real time with custom MATLAB (MathWorks, Natick, USA) software. Power spectrum density (PSD) was computed on the detrended data, weighted by a symmetric Hamming window (length = 250). PSD was calculated as the squared absolute value of the power spectrum output for the defined frequency using the MATLAB FFT function.

#### 2.3.2 Calibration

The calibration task (performed during the premovement assessment) had two aims. First, it verified the presence of a beta‐band rhythm (14–31 Hz) and event‐related beta desynchronization (ERD) over contralateral sensorimotor cortex (electrode C3). This was assessed by computing the mean PSD during the initial eyes‐open resting period to confirm a peak within the beta range and by examining beta power during correct Go trials to confirm the expected desynchronization following the IC.

Second, the calibration phase was used to estimate two participant‐specific parameters required for the ENF phase: the peak beta frequency (fmaxβ) and the individual beta range (Rβ). The peak frequency was computed offline by contrasting PSD during movement‐related beta desynchronization (MRBD; 0–1 s post‐IC) with PSD during baseline (−2 to 0 s pre‐IC) within the nominal beta band (14–31 Hz). The frequency showing the largest difference was selected as fmaxβ. The individual beta range was then defined as Rβ = fmaxβ ± 5 Hz and used during the ENF phase to compute beta desynchronization and synchronisation thresholds (Figure 1).

**FIGURE 2:**
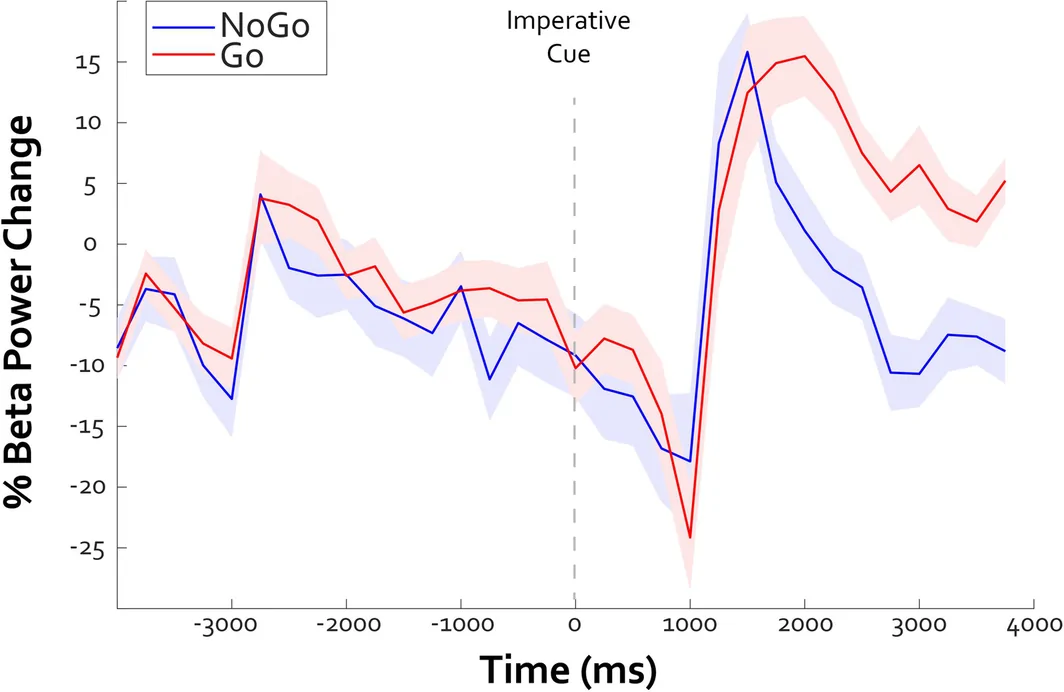
Time course of averaged beta power change across all participants during the Go/No‐go task of the calibration stage. Shaded bands indicate the standard error of the mean for each condition. Data showed here are time‐locked to IC onset, and averages were computed from the saved beta power time courses calculated every 250 ms. Note that the initial beta dip is probably due to the pre‐cue onset.

To verify that the online computations captured the expected beta dynamics, the computed beta‐power time series were time‐locked to trial onset and inspected for the characteristic beta desynchronization following the IC (Figure 2). The raw EEG data were subsequently analysed as described in the section *Analysis of Neurofeedback and Performance*.

#### 2.3.3 Neurofeedback Presentation

Visual stimuli were generated in real time using Psychtoolbox (Brainard 1997) plugin for MATLAB. In each trial of the real‐ENF task, a yellow bar was presented at the centre of the screen. Beta‐band power was estimated from the C3‐Cz EEG signal, with PSD computed every 250 ms using a 500‐ms sliding window.

For each trial, beta ERD and ERS thresholds (thβD and thβS) were calculated from the baseline period using the participant's specific beta range (Rβ; fmaxβ ± 5 Hz). The median PSD within Rβ was calculated across baseline time points, and the interquartile range (IQR) was used to define thresholds: thβD = median − 0.01 × IQR and thβS = median + 0.01 × IQR.

During each trial, bar position was updated every 250 ms based on current beta power. If beta power fell below thβD, the bar moved 10 pixels to the left; if it exceeded thβS, it moved 10 pixels to the right. If beta power remained between thresholds (thβD ≤ β ≤ thβS), the bar moved five pixels toward the centre, ensuring that in the absence of sustained beta modulation, the bar gradually returned to its default position. This design encouraged participants to maintain continuous engagement with the task. The bar position was reset to the centre at the beginning of each trial.

In the sham‐ENF condition, bar movement followed the same algorithm but was driven by replayed EEG data recorded during the calibration phase and was therefore unrelated to the participant's ongoing brain activity. Each session lasted approximately 2.5 h, and the two sessions were completed on separate days within 1 week.

### 2.4 Analysis

#### 2.4.1 EEG

EEG data were analysed offline using the EEGLab (Delorme and Makeig 2004) (https://github.com/sccn/eeglab) plugin for MATLAB. Data from the real‐ENF and sham‐ENF tasks were epoched into trials, based upon the pre‐cue onset (for ENF analysis) and IC marker (go/No‐Go analysis). These epochs included 10 s prior to the cue onset (baseline) and 30 s after the cue onset (ENF trial). The epochs were visually inspected for artefacts, and noisy trials were manually rejected. Subsequently, epochs were band‐pass filtered (basic FIR filter new) based on the individual frequency ranges identified during calibration (14–31 Hz). A notch filter was deemed unnecessary as the band‐pass thresholds were below the mains frequency interference (50/60 Hz). A time‐frequency analysis was conducted to analyse the ENF trials using the short‐time Fourier transform that used a Hanning window with a size of 2 s with a 50% overlap. The power spectral density was computed and averaged across ERD and ERS trials. Offline analysis also examined relative beta, which was calculated as the absolute power of beta during an ENF trial (measured 30 s after cue onset) relative to the absolute power of beta during the baseline period (measured 10 s prior to cue onset).

#### 2.4.2 Neurofeedback Performance

Directional accuracy was used to assess neurofeedback success; directional accuracy calculated the number of times beta power changed in the correct direction within an ENF trial. Increasing beta trials and decreasing beta trials will be referred to as up trials and down trials respectively. For up trials, success was defined as an increase in power relative to baseline. For down trials, success required a decrease relative to baseline. Directional accuracy was expressed as a percentage of 1‐s time points within the 30‐s ENF trial (excluding the 4‐s period prior to cue onset) that showed modulation in the correct direction.

Participants were classified as responders or nonresponders based on their directional accuracy outcomes in the real condition. For each participant, directional accuracy was calculated separately for up and down trials. Participants were classified as responders if they achieved a directional accuracy of 60% or greater. Following this classification, group‐level analyses were conducted separately for directional accuracy. Participants were grouped into four categories based on trial direction and responder status: up responders, up nonresponders, down responders and down nonresponders.

#### 2.4.3 Perceived Neurofeedback Task Difficulty

The verbal responses regarding the difficulty of moving the bar were analysed using content analysis; this is a qualitative method that identifies patterned meaning across a dataset, using codes to identify common ‘themes’ that summarise main points explicitly presented in the data and counted to describe their frequency. Themes were categorised according to group condition (i.e., real or sham).

## 3 Results

### 3.1 Participants' Responder Status

Analysis confirmed fmaxβ values within the 13–30 Hz band for all 15 participants (median = 18 Hz [IQR = 17.5–20 Hz; range = 16.5–27.0 Hz]), with corresponding individual Rβ defined accordingly. For the down trials, 13 participants were responders (87%). For the up trials, three participants were responders (20%). Two participants were responders for both up and down trials (13%). This shows a considerable asymmetry in the ability to modulate beta activity in different directions. All participants requested suggestions for ENF strategies after the first block, and therefore, no subgroup analysis examining the effect of strategy provision on ENF performance was possible.

### 3.2 The Extent to Which ENF Enables Bidirectional Control of Sensorimotor Beta Oscillations

**FIGURE 3:**
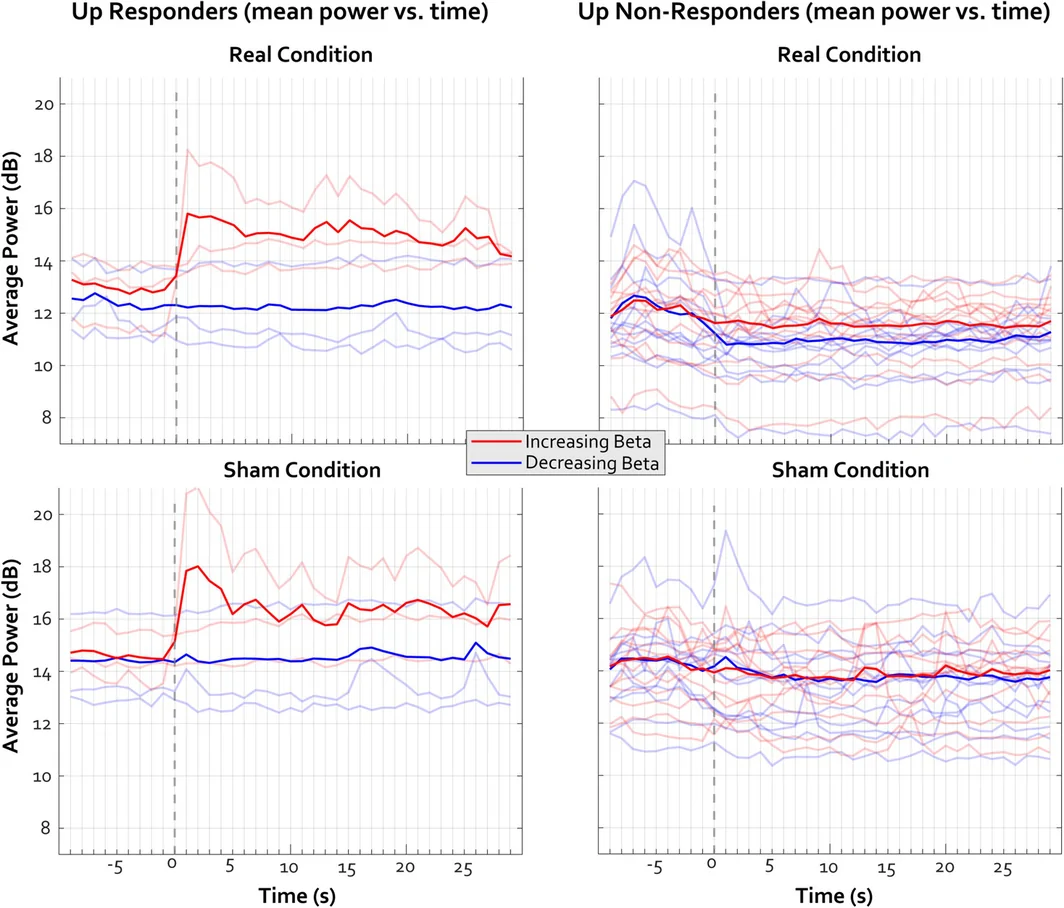
Time–frequency beta plot showing 10 s prior to neurofeedback stimuli onset and 30 s of the neurofeedback trial for up responders (left) and nonresponders (right); solid red and blue lines show the mean, whereas the faded red and blue lines show individual participant data; dashed vertical line indicates onset of the yellow bar, that is, the neurofeedback stimulus.

**FIGURE 4:**
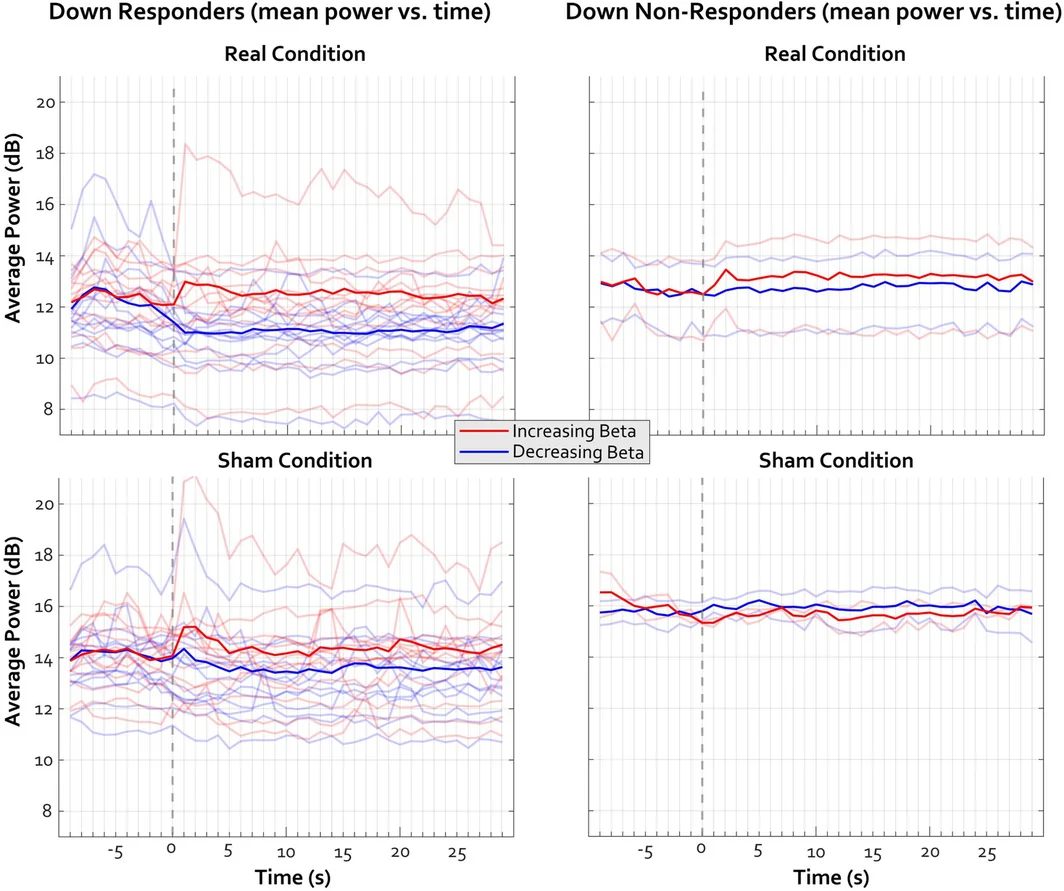
Time–frequency beta plot showing 10 s prior to neurofeedback stimuli onset and 30 s of the neurofeedback trial for down responders (left) and nonresponders (right); dashed vertical line indicates onset of the yellow bar, that is, the neurofeedback stimulus.

Figures 3 and 4 show how beta changed within an ENF trial comparing responders and nonresponders for each direction, and between the sham and real conditions.

Table 1 shows the mean values for directional accuracy. Wilcoxon signed‐rank tests were conducted that compared the up and down trials in the real condition and compared the real and sham conditions. These results can be seen in Table 2.

**TABLE 1:** Mean values for directional accuracy at group level.

Group	Real up trial	Real down trial	Sham up trial	Sham down trial
Down responders	0.51%	0.64%	0.44%	0.61%
Up responders	0.83%	0.55%	0.67%	0.47%
Down nonresponders	0.67%	0.48%	0.53%	0.42%
Up nonresponders	0.56%	0.51%	0.39%	0.52%

**TABLE 2:** Wilcoxon signed‐rank test for directional accuracy at group level.

Group	*z*‐value up versus down trials (real condition)	*z*‐value real versus sham conditions for the corresponding group direction
Down responders	−3.53*	0.99
Up responders	4.37*	3.48*
Down nonresponders	3.16*	1.28
Up nonresponders	0.79	1.55

* *p* < 0.000.

For down responders, both sham (0.61%) and real (0.64%) down trials have similar outcomes (mean difference 0.3%). This may suggest a lack of condition specificity in beta modulation, perhaps due to an effect of real and sham condition order. Therefore, the effect of condition order was examined to provide further clarification, which included both up and down trials. A Wilcoxon signed‐rank test indicated that directional accuracy during real ENF was significantly higher than during sham ENF when participants received the sham condition first (*Z* = 3.80, *p* < 0.001). When participants received the real condition first, the difference in directional accuracy between real and sham ENF was not statistically significant (*Z* = 1.19, *p* = 0.233). This suggests that there is a transfer learning effect, where participants who received the real condition first transferred their beta modulation learning to the following sham condition.

### 3.3 Exploration of ENF Performance and Learning Mechanisms

The exploration of ENF performance and learning mechanisms included examinations of (1) ENF performance changes within a session using directional accuracy, (2) ENF learning persisting beyond a 30s trial (i.e., carryover analysis), and (3) cumulative beta changes within a session (i.e., beta drift analysis).

#### 3.3.1 ENF Performance Changes Within a Session

Changes in ENF performance were assessed using the averaged directional accuracy across participants according to the 5‐min blocks used to divide the session. As a reminder to the reader, each session consisted of six 5‐min blocks of ENF trials. The first three blocks were either up or down trials, and the next three blocks were trials in the opposite direction. Analyses were conducted according to direction of beta control and real or sham condition. Table 3 showcases how responder status changes for each 5‐min block during the real condition, and Table 4 showcases the directional accuracy changes for each block, where responders and nonresponders are identified from responder status shown in Table 3. The sham condition was not examined because the purpose of this analysis was to examine within‐session learning effects during active ENF control.

**TABLE 3:** Responder status changes within a session per 5‐min blocks (real condition).

Direction	Block 1 responders (%)	Block 2 responders (%)	Block 3 responders (%)
Down trials	12 (80)	13 (87)	13 (87)
Up trials	2 (13)	6 (40)	4 (27)

**TABLE 4:** Mean value directional accuracy changes within a session per 5‐min blocks (real condition).

Group	Block 1	Block 2	Block 3
Down responders	0.73%	0.75%	0.76%
Up responders	0.80%	0.77%	0.79%
Down nonresponders	0.52%	0.44%	0.41%
Up nonresponders	0.32%	0.31%	0.37%

Responder status minimally varies across blocks for down trials; however, this status varies more considerably for up trials. Responders increased during Block 2 up trials and then decreased in Block 3. Responder's directional accuracy seemed to minimally vary across blocks for both down and up trials; however, directional accuracy varies more for nonresponders. Down nonresponders' directional accuracy decreased as blocks continued, whereas up nonresponders peaked during Block 3.

To better understand these changes, the verbal responses regarding perceived ENF task difficulty were examined and revealed the following themes:

- Real Condition
  ○ Moving the bar left was easy (*n* = 12, 80%).
  ○ Moving the bar right was hard (*n* = 13, 87%).
  ○ The task became harder as time went on (*n* = 9, 60%).
  ○ I became tired as time went on (*n* = 15, 100%).
  ○ The task became easier after the first break (*n* = 12, 80%).
- Sham Condition
  ○ Task was generally hard (*n* = 12, 71%).
  ○ Moving the bar left was easier than the right, but still hard (*n* = 5, 33%).
  ○ I became tired as time went on (*n* = 15, 100%).

The real condition theme indicates that participants found moving the bar left (i.e., decreasing beta) was easy while moving the bar right (i.e., increasing beta) was challenging. Gradually, the task became harder as time went on and participants become more tired, indicating task fatigue or gradual disinterest in the task. Interestingly, participants found the task easier after the first block, indicating they became acquainted with the task or perceived to become more successful after getting used to the task. The sham condition themes indicate that participants' task difficulty perceptions were mostly not related to the direction of the bar and was a generally difficult task.

Taken together, the above findings indicate that ENF performance varies across the duration of a single session, which differs according to the direction of beta modulation and impacted by cognitive processes of identifying a successful mental strategy, task engagement and fatigue rather than a fixed responder status.

#### 3.3.2 Carryover Analysis

A carryover analysis was conducted to check whether the average beta power during the final 5 s of an ENF trial influenced the subsequent baseline and first 5 s of the subsequent ENF trial. Correlations were calculated between end‐of‐trial beta power and the subsequent baseline, and end‐of‐trial beta power and the beginning of the subsequent ENF trial. Linear regressions were also performed to estimate the slope of any carryover effect.

**FIGURE 5:**
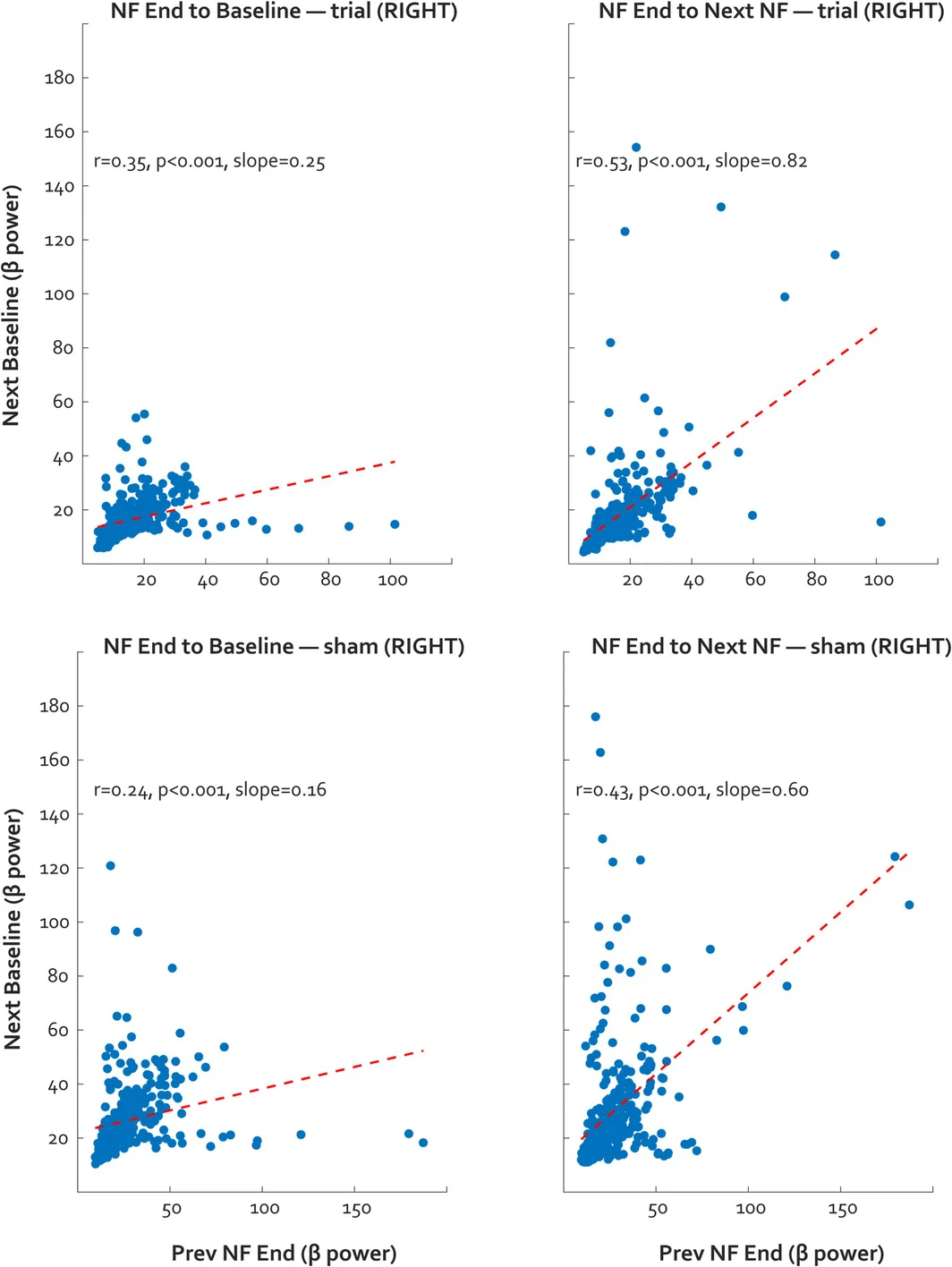
Carryover scatter plot for up trials.

**FIGURE 6:**
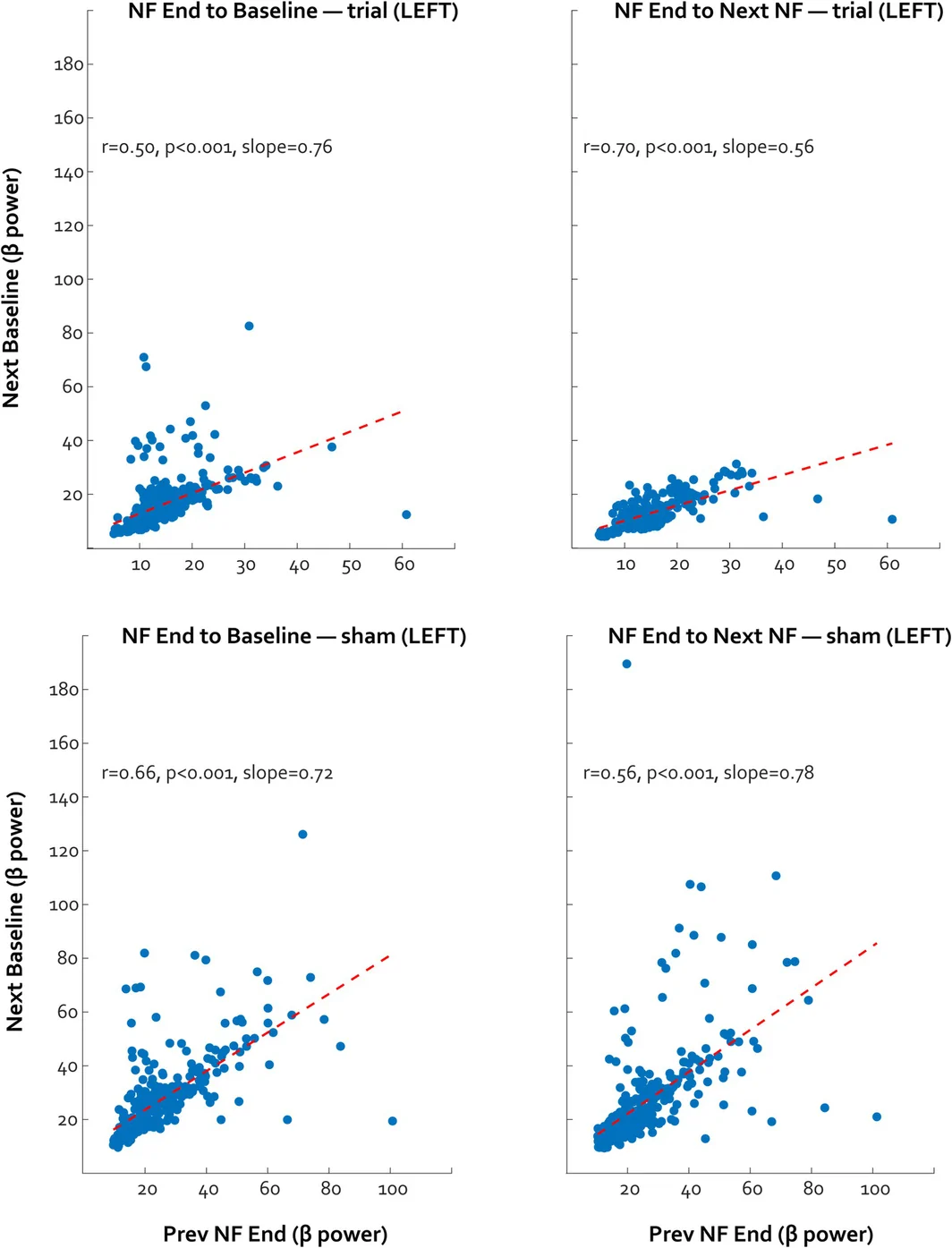
Carryover scatter plot for down trials.

The carryover analysis is shown in Figures 5 and 6, which show a significant carryover effect across all conditions. Stronger effects are observed for the down trials and real conditions. This suggests that participants may have continued their modulation strategies from the previous trial into the baseline and the subsequent trial. This is supported by the earlier examination of effect of order that showed transfer of learning if participants engaged in the real condition first.

#### 3.3.3 Beta Drift Analysis

Trial‐level beta drift was examined to determine whether within‐trial beta control and the carryover effect contributed to cumulative changes in beta power within a session. Although Figures 3 and 4 show that participants modulated beta power in the instructed direction within a 30‐s trial, a small drift in the opposite direction was observed across each 5‐min block. For up trials, responders' beta power began high and declined across the block (mean change = 0.85 dB; SD = 0.73 dB). Conversely, for down trials, responders' beta power began low and increased across the block (mean change = 0.24 dB; SD = 0.13 dB). These trends were observed only in the real condition, did not reach statistical significance and were absent in nonresponders. This suggests that while responders can modulate beta power accurately within a trial, this ability does not translate into sustained, cumulative beta changes across a session. All participants verbally reported fatigue after each block, which may contribute to this drift pattern. Taken together with the carryover outcomes, these findings suggest that the effects of a single ENF session are transient and effort dependent. Multiple training sessions may therefore be required to elicit more stable ENF learning or plasticity.

## 4 Discussion

This study examined ENF performance to determine the extent to which healthy individuals can achieve bidirectional control of sensorimotor beta activity. Thirteen participants successfully decreased beta activity, whereas only three successfully increased it. This asymmetry suggests that control in each direction may reflect distinct ENF abilities, such that bidirectional control cannot be assumed from successful control in only one direction; this concurs with previous research (Enz et al. 2022) that targeted the right inferior frontal cortex over multiple training sessions. One possibility is that beta upregulation is less intuitive or more cognitively demanding than suppression (Anil et al. 2024). However, the findings may also point to mechanistic barriers to beta enhancement, such that voluntary increases in beta power are less readily achievable in this population. This raises the possibility that the neural generators underlying spontaneous beta activity and post‐movement beta synchrony are differentially accessible to cognitive modulation with ENF. Under this account, the relative difficulty of beta upregulation may reflect not simply poor learning or task performance but a more fundamental constraint on the voluntary engagement of neural processes that support increased beta synchrony. This interpretation is consistent with the functional association of elevated motor beta activity with movement inhibition and with the possibility that its modulation depends, at least in part, on broader cortico‐subcortical dynamics.

Importantly, successful ENF control within individual trials did not translate into sustained cumulative changes across the session. Rather than indicating unsuccessful training, this may suggest that effective ENF in this context is better characterised by reliable trial‐by‐trial modulation than by progressive absolute shifts in beta power over time. The observed drift may still reflect fatigue, declining task engagement, or homeostatic constraints on beta activity that limit sustained modulation within a single training session. It also suggests that responder status is not fixed but is influenced by cognitive factors such as the identification of an effective mental strategy, task engagement and fatigue. This interpretation is consistent with previous studies highlighting the importance of behaviour, perceptions and learning in shaping ENF performance (Anil et al. 2024; Kober et al. 2013; Anil et al. 2022; Chikhi et al. 2023; Han et al. 2023). Recent work has therefore begun to adopt adaptive approaches to ENF task difficulty in order to better match training demands to individual ability (Li and Song 2025), reinforcing the need for more personalised ENF protocols. Consistent with this, Bazzana et al. (2022) also called for robust evaluation of personalised neurofeedback approaches to optimise therapeutic effectiveness.

A small, nonsignificant drift in the opposite direction was observed in responders during real ENF, suggesting that sustained modulation may be constrained by fatigue or homeostatic regulation of beta activity. Although participants showed transient within‐trial control, this did not accumulate into sustained session‐level physiological change. At the same time, significant carryover and order effects indicate that participants acquired modulation strategies that transferred across trials and conditions. This supports a distinction between strategic learning and physiological adaptation, the latter of which may require repeated sessions to emerge. Consistent with this, Markiel et al. (2025) found that a 3‐day interval optimised retention of neurofeedback learning, whereas Min et al. (2023) reported sustained physiological change with weekly training. Additionally, Enz et al. (2022) also reported that successful beta modulation was observed during ENF performance but did not persist during subsequent resting‐state recordings (*n* = 44). This is particularly interesting because their study involved a larger sample and repeated training over six sessions, yet still found no evidence of lasting offline changes in beta activity. Early ENF learning may be driven predominantly by the acquisition of cognitive strategies that enable transient online modulation, whereas persistent physiological adaptation (if it occurs at all) follows a different timescale and may require different learning conditions. Future work should investigate the training duration and conditions required for transient modulation to develop into sustained neurophysiological change.

These findings highlight a central issue in ENF research: Success depends on how and when it is measured. In the present study, responder status varied according to whether performance was assessed at the level of individual trials, 5‐min blocks or the full session, whereas directional accuracy, carryover effects and cumulative beta drift captured different aspects of ENF behaviour. This suggests that ENF success cannot be defined by a single metric, but instead reflects multiple processes operating over different timescales, including transient task‐dependent modulation, strategic learning and longer‐term physiological adaptation. This view is consistent with recent work advocating a dynamic, non‐binary approach to ENF performance (Janssen et al. 2017; Schönenberg et al. 2017) and warning that rigid group‐level classifications may obscure meaningful individual learning (Kuznetsova et al. 2023). The present findings also raise methodological concerns for ENF study design, as transfer of learning into sham conditions suggests that crossover designs may be problematic without adequate washout periods.

Another important consideration is that participants were initially encouraged to discover their own ENF strategies. Although this approach was chosen because the influence of explicit strategy instruction on neurofeedback learning remains uncertain, the timing of strategy provision may nevertheless have contributed to the trends seen in our findings. Future studies should systematically evaluate the impact of the timing and content of ENF instruction to determine its contribution to ENF performance.

In the context of motor rehabilitation and Parkinson's disease, these findings suggest that ENF may support moment‐to‐moment suppression of beta oscillations, but that single‐session training is unlikely to induce stable tonic changes in beta dynamics. However, sustained reduction in beta synchrony may not be the most meaningful therapeutic target. A more relevant outcome may be the capacity to produce reliable, reactive modulation when required, as this form of dynamic control is likely to be more important for effective movement. Previous work has reported positive associations between ENF performance and improvements in motor symptoms in Parkinson's disease (Simon et al. 2021). However, the EEG features proposed to underlie these effects remain inconsistent. For example, some studies have focused on sensorimotor rhythm as the relevant target (Han et al. 2023), whereas others have argued that beta bursts may be more informative in this context (He et al. 2020; Wessel 2020). It is also possible that movement‐relevant ENF effects depend less on any single beta feature than on the broader capacity to regulate cortical beta dynamics (Anil et al. 2024). We therefore suggest that the therapeutic mechanisms of ENF in Parkinson's disease may be better understood by examining the individualised and dynamic nature of ENF learning and performance. Critically, future work must also address ecological validity. Most ENF studies are conducted in constrained desktop settings, whereas motor impairment in Parkinson's disease is expressed through whole‐body, gait and lower limb dysfunction. Translation will therefore require approaches that test whether ENF can support adaptive beta control in more functionally relevant, real‐world motor contexts.

### 4.1 Strengths and Limitations

Key strengths of this study are the inclusion of a blinded sham control condition, and the comparison of both upregulation and downregulation of sensorimotor beta oscillations. This study also evaluated ENF performance across within‐trial modulation, within‐session learning, carryover effects and beta drift. This approach provides a more nuanced understanding of neurofeedback learning processes and highlights how different analytical perspectives can produce different interpretations of performance. Finally, this study incorporated participant‐reported experiences of task difficulty and fatigue, which helped contextualise the neurophysiological findings.

Some limitations should also be noted. The relatively small sample limits the findings' generalisability. The findings should be considered preliminary. However, the findings are consistent with findings reported in a larger similar study (*n* = 44) mentioned above investigating beta control over the right inferior frontal cortex (Enz et al. 2022), providing convergent evidence for the observed effects found in this study and supporting their potential reproducibility. Nevertheless, replication in a larger randomised controlled trial is required. In addition, order effects between real and sham conditions suggest that participants may transfer learned strategies across conditions. Although this provided valuable insight into ENF learning mechanisms, it also highlights a methodological limitation of crossover sham designs, where prior exposure to real ENF may influence subsequent control conditions. The present study explores the ability of healthy control participants to modulate their motor cortical beta power. Although there are clear opportunities to translate these approaches to related pathological conditions such as Parkinson's, it should be noted that this raises further considerations. Firstly, it is unclear whether the mechanism of altered beta signatures in Parkinson's are consistent with those modulated here. Secondly, the presence of symptoms such as tremor, with continuous and unpredictable movement, presents additional technical challenges for applying neurofeedback approaches.

### 4.2 Future Research

The present findings provide a useful framework for understanding ENF performance by combining blinded sham comparison with a multi‐timescale analysis of modulation, carryover and drift. At the same time, interpretation should remain cautious given the modest sample, single‐session design and evidence that learned strategies transferred between conditions. These findings point clearly toward the next phase of ENF research: larger multi‐session studies, more careful control designs and analysis frameworks that distinguish transient task performance from longer term physiological adaptation. For rehabilitation, this distinction is particularly important, as the most meaningful outcome may not be a sustained tonic shift in beta activity, but the restoration of reliable, reactive modulation when required. Future work should therefore test whether such dynamic control relates to clinical benefit and, critically, whether it can be translated beyond constrained desktop paradigms into more ecologically valid settings that capture the whole‐body and lower limb impairments characteristic of neurological disorders such as Parkinson's disease. Finally, future research should evaluate ENF performance metrics in Parkinson's disease populations to determine their generalisability to clinical populations.

## 5 Conclusion

This study showed that early ENF success depends critically on how performance is defined and measured. Healthy participants were able to transiently modulate beta activity within individual trials, but this did not translate into sustained change across the session. Carryover effects and learning transfer between real and sham conditions further suggest that early ENF performance primarily reflects strategy‐dependent, effort‐based control rather than stable neurophysiological plasticity. These findings highlight the need for a more precise framework for ENF evaluation, one that separates transient modulation, strategic learning and durable physiological adaptation. This will be essential for refining ENF protocols and improving their clinical relevance. The challenge now is not simply to show that beta can be modulated, but to determine when that modulation is reliable, functionally meaningful and transferable to real‐world rehabilitation.

## Ethics Statements

All subjects gave their informed consent for inclusion before they participated in the study. The study was conducted in accordance with the Declaration of Helsinki, and the protocol was approved by the University of Plymouth's Faculty of Health Research Ethics and Integrity Committee (19/20‐1322).

## Author Contributions

**Krithika Anil:** conceptualization, writing – review and editing, writing – original draft, data curation, formal analysis, investigation, methodology, project administration, visualization, validation. **Stephen Hall:** conceptualization, writing – review and editing, methodology, formal analysis, investigation. **Jennifer A. Freeman:** writing – review and editing. **Jonathan Marsden:** conceptualization, writing – review and editing, methodology, formal analysis, investigation. **Giorgio Ganis:** conceptualization, writing – review and editing, writing – original draft, methodology, software, investigation, formal analysis, visualization, validation, resources.

## Conflicts of Interest

The authors declare no conflicts of interest.

## Supporting information


**DATA S1:** Supporting Information.

## Data Availability

Data available in a publicly accessible repository via the University of Plymouth depository Pure. Code can be made available on request.
